# Few Shot Class Incremental Learning via Efficient Prototype Replay and Calibration

**DOI:** 10.3390/e25050776

**Published:** 2023-05-10

**Authors:** Wei Zhang, Xiaodong Gu

**Affiliations:** Department of Electronic Engineering, Fudan University, Shanghai 200438, China; wzhang20@fudan.edu.cn

**Keywords:** few shot learning, incremental learning, meta-learning, feature replay, prototype calibration

## Abstract

Few shot class incremental learning (FSCIL) is an extremely challenging but valuable problem in real-world applications. When faced with novel few shot tasks in each incremental stage, it should take into account both catastrophic forgetting of old knowledge and overfitting of new categories with limited training data. In this paper, we propose an efficient prototype replay and calibration (EPRC) method with three stages to improve classification performance. We first perform effective pre-training with rotation and mix-up augmentations in order to obtain a strong backbone. Then a series of pseudo few shot tasks are sampled to perform meta-training, which enhances the generalization ability of both the feature extractor and projection layer and then helps mitigate the over-fitting problem of few shot learning. Furthermore, an even nonlinear transformation function is incorporated into the similarity computation to implicitly calibrate the generated prototypes of different categories and alleviate correlations among them. Finally, we replay the stored prototypes to relieve catastrophic forgetting and rectify prototypes to be more discriminative in the incremental-training stage via an explicit regularization within the loss function. The experimental results on CIFAR-100 and *mini*ImageNet demonstrate that our EPRC significantly boosts the classification performance compared with existing mainstream FSCIL methods.

## 1. Introduction

Compared with artificial intelligence, human intelligence possesses a unique ability to learn from only a small amount of data, relying on its special memorizing and reasoning capabilities [1]. Motivated by this observation, current research mainly focuses on training and optimizing model parameters or modifying model structures to enable it to learn from only a few samples, which is denoted as few shot learning [2,3]. The primary objective of few shot learning is to leverage large-scale data of base categories to enhance the generalization ability of models and then obtain discriminative features for new categories with merely few training data. Recent popular few shot learning methods usually employ meta-learning and metric learning to acquire generalizable representations and perform classification. However, in realistic scenarios, both human and artificial intelligence face a similar challenge: without enough memory, previously learned information may be forgotten as knowledge accumulates continually, thus leading to mistakes in reasoning. To simulate this situation, researchers have proposed incremental learning tasks [4], where models are trained to alleviate the forgetting problem to some extent. Few incremental learning, as an integration of few shot learning and incremental learning, has great practical value, but also presents significant challenges. In particular, it is necessary to address the catastrophic forgetting problem [5,6] of prior knowledge from old categories in incremental learning, as well as the overfitting problem of new categories in few shot learning.

The problem of catastrophic forgetting [5,6] in incremental learning is primarily caused by the drift of parameters in the model. This phenomenon can be explained from two perspectives. First, from the local perspective, when updating the weights associated with the old categories by utilizing new category data, minimizing the loss of new categories could result in the performance decline of old categories. Second, from the global perspective, in class incremental learning, the model is trained only on new category data in each incremental stage, and all encountered categories are never trained together, thus causing sub-optimal differentiation of these categories at most after updating the model parameters.

To address this issue, three general kinds of approaches have been proposed. With regard to models, the first kind suggests continually expanding and updating the network structure, such as in [7]. However, it will increase the complexity of the network in each incremental session, which is challenging to implement in practice. With regard to optimization algorithms, regularization constraints on the parameters or features can make models inclined to infer as before, thereby maintaining the performance on old categories, such as in [8,9]. With regard to data, data storage and retrieval of old categories is an explicit way to alleviate forgetting. However, storing original data [10] is limited by the size of memory, and generating data or features [11] requires plenty of training data, which makes it difficult to implement in few shot scenarios. Therefore, in this paper, we store prototype features for each encountered category and then conduct prototype replay and calibration in each few shot incremental stage, which is more practical and effective.

**Figure 1:**
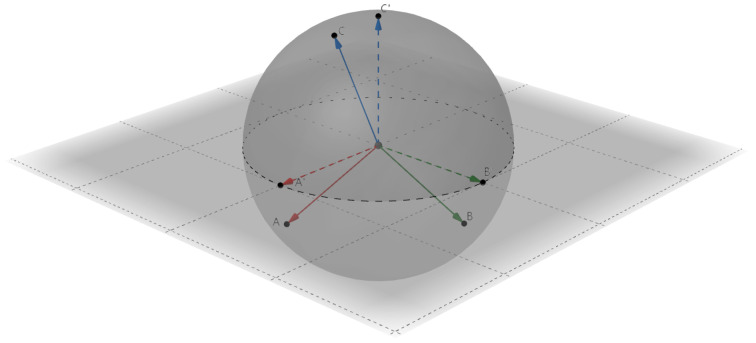
The diagram of prototypes’ distribution in the normalized feature space (3D space as an example). In this unit sphere, three vectors described by directed solid lines of different colors represent three actual prototype features of different categories. Another three vectors described by directed dashed lines of different colors represent the ideal orthogonal situation.

Additionally, we perform pre-training with strong augmentations as well as popular meta-learning to tackle the overfitting problem of new categories in few shot scenarios, thus obtaining discriminative features. With regard to prototypes, as demonstrated in Figure 1, there always exist certain correlations between the actual normalized prototype features in unit sphere unless they are strictly orthogonal, which will cause interference in classification. Generally, two prototype features are more unrelated when their angle is closer to 90 degrees, thus making them more discriminative. However, the dimension of feature space limits the total number of orthogonal vectors that can be achieved; thus, we do not orthogonalize these features. Instead, we make implicit restrictions in the training stage to calibrate the generated prototypes of different categories to make them less correlated so that they are capable of distinguishing each other.

Based on the above analysis, we propose an efficient prototype replay and calibration (EPRC) with three stages to adapt to few shot incremental tasks. For the preliminary, we first utilize the rotation and random mix-up of input data or intermediate features as a strong augmentation in the pre-training stage to promote the backbone to generate strong representations. With limited support samples, novel incremental sessions are quite different from the base session. To alleviate overfitting of these few shot novel categories, we randomly sample a batch of pseudo few shot learning tasks in large-scale base datasets to help the model adapt to few shot scenarios, which we call the meta-training stage. Moreover, a feature projection layer is trained together with the backbone to map the initial prototypes into more discriminative ones. To mitigate the overlap of correlations among prototypes of different categories, we leverage an even nonlinear transformation function to alter the softmax function and then implicitly calibrate these prototypes. Finally, with respect to catastrophic forgetting in the incremental stage, we store prototypes encountered before and replay them in the third incremental-training stage to further assist distinguishing among all the prototypes. We also conduct binarization for prototypes (which can be regarded as weights of a classifier) to simplify the model, thereby reducing the risk of overfitting. With an explicit regulation in loss function for fine-tuning the projection layer, the stored prototypes can be refined better. To validate the effectiveness and rationality of three stages in our EPRC, we conduct experiments on public datasets, i.e., CIFAR-100 and *mini*ImageNet. Our contributions can be summarized as follows:

- We design a holistic three-stage training scheme for few shot class incremental learning, which can be applied as a generalized pipeline to enhance the quality of prototypes in a step-by-step way.
- To avoid overfitting, we introduce a pre-training stage with strong augmentations and a meta-training stage with implicit prototype calibration which reduce overlap among features of different categories.
- To mitigate catastrophic forgetting, we perform prototype storage and replay together with an explicit prototype rectification by leveraging both base and novel knowledge in the incremental-training stage.
- The experimental results on CIFAR-100 and *mini*ImageNet datasets demonstrate that our EPRC significantly boosts the classification performance compared with existing mainstream FSCIL methods.

## 2. Related Work

### 2.1. Few Shot Learning

The characteristic of few shot learning lies in the limited training data, which causes a lack of prior knowledge. To compensate for this deficiency, data augmentation or complex task augmentation [12,13] can be performed to enable the model to learn richer prior knowledge. For example, ContrastNet [12] simultaneously performs instance-level and task-level data augmentation to avoid overfitting caused by insufficient data during training. Utilizing contrastive learning, it learns more discriminative features in similar augmented samples and tasks. Similarly, IEPT [13] expands the sampled original task to four augmented tasks by rotation, thus improving the generalization ability of the feature extractor. Based on these kinds of augmentation, few shot learning can then be further divided into two categories: metric-learning-based and meta-learning-based.

Generally, the goal of metric learning is to minimize the intra-class distances and maximize the inter-class distances. Metric-learning-based methods such as [14,15,16] usually focus on the relationship between instances. They first map support and query instances into the same feature space, and then predict based on the calculated similarity between their features. Other works have made a series of extensions based on [14,15,16] and have made good progress. AM3 [17] integrates additional cross-modal knowledge (such as semantic representation) into the prototypical network [15]. BiDiLEL [18] learns to project visual embeddings into a latent space to bridge the gap between the visual representation space and the semantic space. DeepEMD [19] explores the relationship between local feature embeddings from different images and searches for the optimal match between the support and query samples. Then it can measure distances by minimizing the cost of matching. From the perspective of labels, SPL [20] explores structural information via unsupervised learning and utilizes selective pseudo-labeling and structured prediction in the target domain without annotated data. ArL [21] comprehensively studies absolute and relative label information to obtain more realistic and accurate relationships between images. Moreover, as a special part of metric learning, methods based on graph neural networks such as [22,23,24,25,26] mainly perform representation learning by iteratively aggregating features from neighboring nodes and updating node or edge features, thus exploring complex relations between features.

Meta-learning-based methods focus on designing optimization steps in the meta-training process to update the model parameters so as to obtain a more generalized model, whose interpretability is relatively strong, according to [27]. As a milestone method, MAML [28] aims to find suitable initial parameters through cross-task training. Based on these initialized parameters, the base learner can quickly generalize well on new tasks with few labeled examples. Inspired by [28], TAML [29] introduces an unbiased task-independent prior on the original model to prevent it from over-relying on meta-training tasks. CAML [30] designs a parameterized conditional transformation module based on label structure, which can modulate the representation of the base learner according to the current task. With regard to computational cost, LEO [31] maps the parameters in the high-dimensional space to a low-dimensional latent space through a trained encoder to accelerate the speed of model learning and adaptation. MetaOptNet [32] combines a QP solver with a linear classifier to further reduce the computational cost. Unlike [28], BOIL [33] freezes the classifier during the inner optimization process of meta-training to enable the model to have a stronger generalization ability towards variable representations, which is essential for domain-agnostic tasks.

### 2.2. Few Shot Class Incremental Learning

Traditional neural networks are typically trained on a fixed large-scale dataset of pre-defined classes and evaluated on these known classes. However, in dynamic and open real-world applications, new classes emerge constantly, and thus the model needs to continually improve its discriminative ability to handle these new classes. Few shot class incremental learning (FSCIL), which sequentially learns from a series of datasets containing different classes with limited data, is distinct from conventional classification methods in that it can classify all encountered classes simultaneously. In few shot class incremental learning, when the model updates its parameters leveraging data of new classes, it often suffers from the overfitting problem of old classes of insufficient training data catastrophic forgetting. These two problems limit the practical application of conventional classification models in open environments.

Firstly, to deal with the overfitting problem in FSCIL, recent works set out from either data augmentation or feature refinement. With regard to data augmentation, Ref. [34] trains a generator in the base session and generates samples for novel classes as a supplement of training data. Another kind of augmentation [35,36,37] is similar to meta-learning (or pseudo incremental learning [36]), which samples plenty of few shot tasks (episodes) in base session to simulate the few shot incremental scenarios. Then they can force features adaptive to various randomly simulated incremental processes and then enhance model’s generalization ability by training on these episodes. With regard to feature refinement, TOPIC [38] calibrates the feature representation for novel classes by adapting Neural Gas(NG) to new training samples. SPPR [35] explicitly explores the relationships of different classes and then strengthens the expressiveness of novel classes.

Secondly, to tackle the catastrophic forgetting problem, there are mainly three kinds of methods: network-structure-based, regularization-based, and replay-based. Network-structure-based methods continuously expand the network structure in incremental stage, which makes it challenging to apply them in a continuous long sequence process. Regularization-based methods can be further divided into parameter-based and distillation-based ones. Parameter-based methods prevent the model from forgetting old knowledge by regularizing the parameters. For example, EWC [39] uses Fisher information to measure the importance of different parameters in the model for old classes and then optimizes unimportant parameters, while freezing or constraining important parameters during class incremental stage. Distillation-based methods [8,9] ensure consistency between the model’s output for specific inputs before and after updating the parameters by designing the loss function. In addition to distilling sample features, methods such as TPCIL [40] impose constraints on the similarity of neighboring nodes and explicitly penalize changes in such relationships in the loss function after parameter updating. Apart from that, the most intuitive way to address the problem of forgetting old knowledge is to store it. When encountering new classes, replay-based methods replay old knowledge by storing real samples of old classes, generated samples or features. Since retaining real data is influenced by the strict storage requirements, data sampling and storage are needed (e.g., iCaRL [10]). The impact of data security has also spurred research on generator-based methods. In contrast, saving and replaying representative features are more advantageous than real data in terms of computational time and storage space. Therefore, many recent works [11,34,36,41,42,43] as well as our method are based on feature replay.

### 2.3. Prototype Calibration in Metric Learning

In metric learning, it is crucial to obtain discriminative instance and prototype feature representations. However, due to the insufficient training data in few shot learning, directly obtaining reliable prototypes becomes challenging, making prototype calibration indispensable. AM3 [17] introduces extra cross-modal knowledge into the prototypical network by utilizing GloVe [44] to extract word embeddings for semantic labels and adaptively combining visual features and semantic features in a convex way to construct new prototypes. BD-CSPN [45] uses pseudo-labeling to mitigate intra-class bias. Additionally, it computes an offset term to compensate for the bias between different sets of support and query categories. CEC [36] adaptively updates weights of the classifier (another form of prototypes) in the constructed graph. ICI [46] iteratively selects unlabeled samples with high confidence and adds them to the support set for data augmentation after assigning pseudo-labels. Then it updates the class centers for the next iteration. LR+DC [47] computes the statistical information of base classes and transfers it to the support set composed of new classes. Specifically, it selects several nearest neighbors of base class samples for each novel class sample and introduces them into the calculation of new class statistics. Similarly, Ref. [35] dynamically measures the relationship between new class samples and old base classes to facilitate prototype updating during meta-training and meta-testing. PT+MAP [48] also iteratively estimates new prototypes and updates them proportionally to obtain more accurate class centers during the assignment of the query set and its corresponding classes. Following [46,48], ILPC [49] predicts pseudo-labels for query samples and selects a portion of cleaned queries with high quality to expand the annotated datasets.

## 3. Materials and Methods

### 3.1. Problem Definition

**Figure 2:**
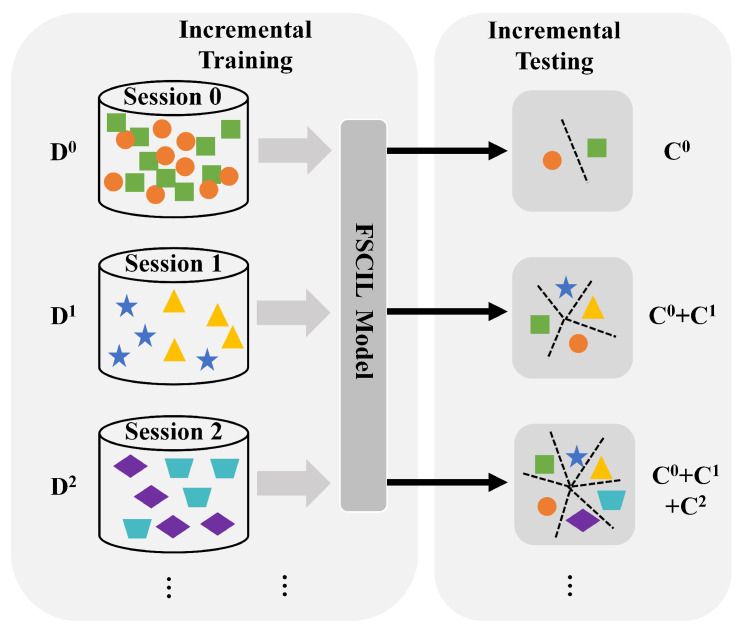
Pipeline of few shot class incremental learning (FSCIL).

Different from few shot learning (FSL), few shot class incremental learning (FSCIL) aims to sequentially learn novel knowledge from new classes with merely few annotated samples and simultaneously alleviate the forgetting of old classes. As shown in Figure 2, given a sequence of training datasets $\left\{D^{0},D^{1},\ldots ,D^{t},\ldots \right\}$ in session {0,1,…,t,…}, where $D^{t}={\left\{\left(x_{i}^{t},y_{i}^{t}\right)\right\}}_{i=1}^{|D^{t}|}$ contains training samples and its corresponding labels, the FSCIL model is trained progressively. Supposing that the category set of session *t* is $C^{t}$, there is no overlap between category sets of different sessions, i.e., i,j∈{0,1,2,…,t,…} and i≠j, $C^{i}\cap C^{j}=\emptyset$. More concretely, $D^{0}$ of base classes in session 0 is usually a large-scale dataset, where model is pre-trained. When t≥1, the form of incremental session *t* is a typical *N*-way *K*-shot task (e.g., 5-way 5-shot). Each task consists of *N* novel classes with *K* labeled training samples. The scale of these few shot tasks is extremely small, i.e., $NK\ll |D^{0}|$. Similar to incremental learning, $D^{t}$ is only accessible to the model in session *t* in FSCIL. Moreover, the model could also leverage a small amount of stored information from previous sessions. After each incremental-training stage, the model will be evaluated comprehensively on the test set of all encountered classes ever in an inductive way, i.e., $C=\cup_{i=0}^{t}C^{i}$.

### 3.2. Overview of EPRC

**Figure 3:**
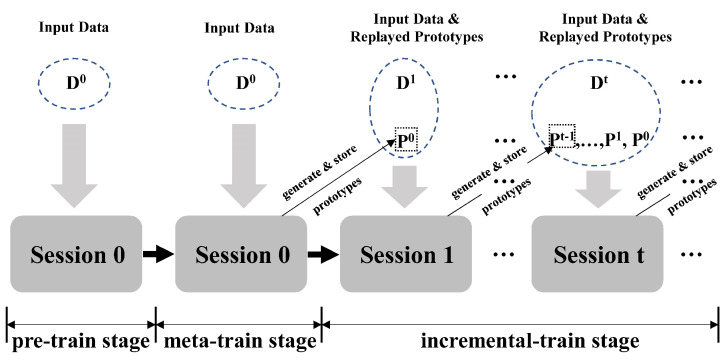
The pipeline of EPRC. Circles with a blue dashed line denote available data in each session. Rectangles with a black dashed line represent the generated and stored prototypes of newly encountered classes in the previous session.

In this section, we elaborate on the details of the efficient prototype replay and calibration (EPRC) method for few shot class incremental tasks. As shown in Figure 3, EPRC consists of three stages: pre-training, meta-training, and incremental-training stage. The purpose of the pre-training stage is to initially obtain an effective feature extractor. In the meta-training stage, EPRC learns to obtain generalized parameters through a series of randomly sampled few shot tasks, thus generating distinguishable prototype features and then being able to quickly adapt to new classes with few annotated samples in the incremental stage. In the incremental-training stage, based on the strong backbone obtained from the first two stages and the replayed prototypes, the parameters of a projection layer are further optimized via novel support samples. By and large, the pre-training and meta-training stages consider only base classes with plenty of training data. When encountering novel classes with few annotated data, incremental training serves as a supplement and refinement for the first two stages to make prototypes of both base and novel classes more discriminative from a holistic point of view by studying the relationships of both the base and novel prototypes. The details of each stage will be presented in the following sections.

#### 3.2.1. Efficient Pre-Training Strategy

During the pre-training stage, following [48,49], we first utilize the large-scale dataset $D^{0}$ to train the backbone on the standard classification task with rotation as an augmentation so as to obtain discriminative features. The standard prediction loss on the base class dataset $D^{0}$ is as follows:

(1) $$L_{pre}=\frac{1}{|D^{0}|}\sum_{x\in D^{0}}L\left(C\left(f\left(x\right)\right),y\right),$$

where L represents the cross-entropy loss, *C* represents a linear classifier, f(·) represents the feature extractor, and *y* represents the label of the input sample *x*.

**Figure 4:**
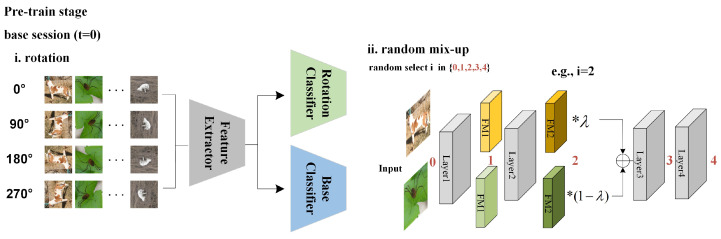
The framework of the pre-training stage of our proposed EPRC for FSCIL. Rotated input images serve as the auxiliary supervision. In random mix-up, a pair of input images or output feature maps of one layer that is randomly selected from the backbone are fused in a proportional way. FM denotes the feature map.

Based on the standard classification task, we rotate the input image *x* by *r* degrees and input both the rotated and original images into the model for training, where $r\in D^{r}=\left\{0^{\circ },90^{\circ },180^{\circ },270^{\circ }\right\}$. The standard classification loss on $D^{0}$ is then formulated as follows:

(2) $$L_{C}=\frac{1}{|D^{r}\left|\right|D^{0}|}\sum_{x\in D^{0}}\sum_{r\in D^{r}}L\left(C\left(f\left(x^{r}\right)\right),y\right),$$

where L represents the cross-entropy loss, *C* is a cosine classifier with softmax, f(·) represents the feature extractor, and *y* is the label of $x^{r}$. Moreover, the rotation loss for self-supervised training is as follows:

(3) $$L_{R}=\frac{1}{|D^{r}\left|\right|D^{0}|}\sum_{x\in D^{0}}\sum_{r\in D^{r}}L\left(C_{R}\left(f\left(x^{r}\right)\right),r\right),$$

where $C_{R}$ is a linear rotation classifier, which aims to classify the rotation angle of input image. As demonstrated in Figure 4, after training the backbone with the sum of classification loss $L_{C}$ and rotation loss $L_{R}$, we further conduct random mix-up for training of feature extractor, which is inspired by [50]. Assuming there are 4 layers in the backbone, considering a pair of different inputs $x_{a}$ and $x_{b}$, we randomly select i∈{0,1,2,3,4} to mix up the *i*-th data in a proportional way, where i=0 represents the initial input image and i>0 represents the output feature map of the *i*-th layer. The specific process is as follows:

(4) $$f_{m,i}=\lambda f_{i}\left(x_{a}\right)+\left(1-\lambda \right)f_{i}\left(x_{b}\right),$$

where λ is sampled from a β-distribution, and $f_{i}\left(\cdot \right)$ denotes the output of *i*-th layer in the backbone. The combined feature $f_{m,i}$ is then fed into the remaining layer of the backbone after layer *i* to generate the final mix-up representations $f_{mix}$. Then we can compute the mix-up loss term for this pair of inputs:

(5) $$L_{m}=\lambda L\left(C\left(f_{mix}\right),y_{a}\right)+\left(1-\lambda \right)L\left(C\left(f_{mix}\right),y_{b}\right),$$

Then the total mix-up loss can be formulated as:

(6) $$L_{M}=\frac{1}{|F_{m}|}\sum_{f_{mix}\in F_{m}}L_{m},$$

where $F_{m}$ represents the set of all features obtained after mixing up. In general, the combination of rotation and mix-up could greatly enhance the generalization ability of our backbone.

#### 3.2.2. Implicit Prototype Calibration in Meta-Training Stage

**Figure 5:**
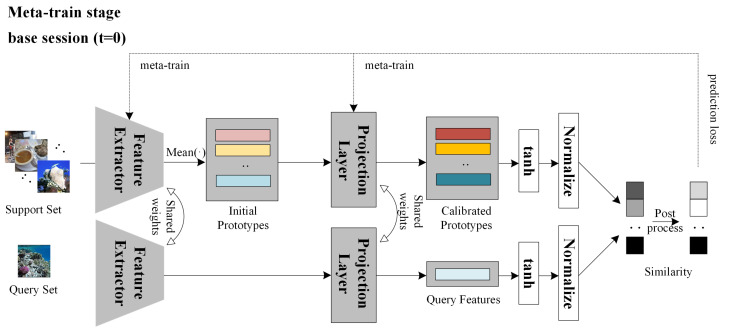
The framework of the meta-training stage of our proposed EPRC for FSCIL. The feature extractor and projection layer are trained together.

The purpose of meta-training is to further optimize the model so that it has good generalization performance on new categories that will be encountered in the incremental stage, and generates robust feature embeddings for input images. To achieve this, in this stage, *K* support samples and *M* query samples are randomly selected for each class in the dataset $D^{0}$ to form a series of few shot tasks, which is a simulation of the few shot incremental process. By meta-training with these randomly sampled few shot tasks, the model can quickly adapt to new categories with few annotated samples. The pipeline of meta-training is demonstrated in Figure 5. First, the pre-trained model is used to extract features from the support samples of each task, and the initial prototypes of each class *j* are calculated as:

(7) $$p_{j}=\frac{1}{K}\sum_{\left(x,y\right)\in D_{S}^{0}}f\left(x\right)\cdot 𝟙\left(y=j\right),$$

where $D_{S}^{0}$ denotes support set in $D^{0}$ and 𝟙(·) is an indicator function. Then a trainable feature projection layer is utilized to calibrate the initial prototypes into more robust and discriminative ones:

(8) $$p_{j}^{\prime }=Proj\left(p_{j}\right).$$

In order to generate more discriminative and robust feature embeddings, the parameters of feature extractor and the projection layer are optimized together in the meta-training stage to improve the calibration ability of the projection layer. For each query sample $x_{q}$ of a specific sampled task, its similarity with each calibrated prototype $p_{j}^{\prime }$ of each class is measured as follows:

(9) $$s_{j}=cos\left(tanh\left(Proj\left(f\left(x_{q}\right)\right)\right),tanh\left(p_{j}^{\prime }\right)\right),$$

where cos(·) represents cosine similarity, and tanh(·) is a nonlinear function to map feature vectors to finite values (−1 to 1). The above formula indicates that the larger the cosine similarity between a query sample and a prototype, the more likely the query sample belongs to the corresponding class *j*. The probability of the query sample belonging to class *j* is then computed:

(10) $$prob_{j}=\frac{\zeta (s_{j})}{\sum_{j=1}^{|C^{0}|}\zeta \left(s_{j}\right)}.$$

$\zeta (s_{j})$ is usually designed to be a monotonically increasing nonlinear function to ensure that the probability increases as the similarity increases. For example:

(11) $$\zeta \left(s_{j}\right)=\zeta_{1}:=e^{\alpha s_{j}},$$

where α is a scale factor.

According to the previous analysis, we hope that features generated for samples of different classes can be less correlated, which means that the cosine value is far away from 1 or −1. Therefore, from this special respect of inter-class difference, an improved $\zeta (s_{j})$ is designed as an even nonlinear function whose curve first decreases and then increases. The form is as follows:

(12) $$\zeta \left(s_{j}\right)=\zeta_{2}:=sigmoid\left(\alpha \left(s_{j}-0.5\right)\right)+sigmoid\left(\alpha \left(-s_{j}-0.5\right)\right).$$

Under this circumstance, for two feature vectors whose angle is close to 0 degree, the prediction results remain unchanged, while for those with angles of 180 degrees, the original prediction results are dissimilar. After the nonlinear transformation of the above formula, their prediction results become similar. After training, the projection layer has the ability to project features into a new space and enhance inter-class dissimilarity. Ideally, the angles between the feature vectors of different classes in this new space are close to 90 degrees (i.e., orthogonal). The loss function in the meta-learning stage is also a standard cross-entropy prediction loss $L_{pre}$.

#### 3.2.3. Explicit Prototype Replay and Calibration in the Incremental-Training Stage

**Figure 6:**
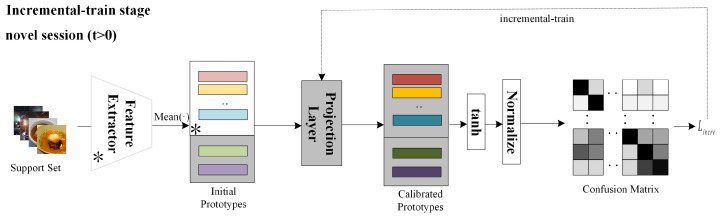
The framework of the incremental-training stage of our proposed EPRC for FSCIL. The feature extractor is frozen and the projection layer is fine-tuned. * represents that the feature extractor is frozen.

After the pre-training and meta-training stages, the prototypes of old categories are stored for replaying in each incremental stage, as shown in Figure 6. Since we can only have access to these replayed prototypes and novel support samples, incremental training serves as a refinement of the feature projection layer. The ideal situation is that old replayed prototypes as well as novel prototypes are as discriminative as needed. However, novel prototypes are generated individually without considering their relations of old prototypes. Therefore, as a supplement of implicit prototype calibration, we conduct metric learning between prototypes of all encountered classes to further calibrate them into more discriminative ones from a holistic point of view. To alleviate overfitting problems in few shot incremental tasks, we also conduct binarizations before computing similarities for all prototypes to simplify the model and make it pay more attention to the angles between prototypes rather than the numerical values of a certain dimension. The loss function for refining the feature projection layer in the incremental-training stage is as follows:

(13) $$L_{inre}=\frac{2}{\left(|C|-1)|C\right|}\sum_{i=1}^{|C|-1}\sum_{j=i+1}^{\left|C\right|}cos\left(tanh\left(p_{i}^{\prime }\right),tanh\left(p_{j}^{\prime }\right)\right),$$

where we compute the mean cosine similarities between different prototypes and then constrain them to make prototypes of different categories more distinguishable. We use gradient descent to optimize the parameters of the feature projection layer in the incremental-training stage Inductive reasoning is conducted in each incremental-testing stage based on the predicted probability of each query sample:

(14) $$\hat{y}=\underset{j}{\arg\max}\;prob_{j}.$$

## 4. Results

### 4.1. Datasets and Experimental Settings

Our experiments are conducted on two widely used benchmark datasets, i.e., CIFAR-100 [51] and *mini*ImageNet [14]. CIFAR-100 [51] contains 100 categories with 600 labeled samples in each category. The original resolution of 32 × 32 pixels makes tasks on it harder and also speeds up inference. *mini*ImageNet [14] is derived from the original ILSVRC-12 dataset [52]. It also consists of 100 classes with 600 labeled instances each. The resolution of all images is 84 × 84 pixels. In few shot class incremental learning, we follow the standard splits and experimental settings used in [36,38] for fair comparisons. Both of the aforementioned datasets containing 100 classes are divided into two parts: 500 training samples and 100 testing samples per class. For training, the first session contains all the 30,000 samples from 60 classes, and each of the following eight incremental sessions will encounter five new classes, with only five samples randomly selected from the 500 labeled data of each class for training in each incremental stage. For evaluation in the *t*-th session, predictions are made for all testing samples from 60+5t seen classes, i.e., there are 100(60+5t) query samples each time.

### 4.2. Implementation Details

In all experiments, ResNet-12 is utilized as a feature extractor for few shot class incremental learning, and the dimension of the output features is 640. In the pre-training stage, the SGD optimizer is used to train the backbone with rotation and mix-up augmentation on $D^{0}$ respectively for 400 and 100 epochs. The learning rate of the pre-training stage is set to 0.01. In the meta-training stage, the feature projection layer maps the 640-dimensional vectors to 512-dimension ones and is trained together with the backbone on 100,000 sampled few shot tasks (each task consists of 60 classes, with 5 support samples and 64 query samples per class for CIFAR-100, and 5 support set samples and 16 query samples per class for *mini*ImageNet). The scale factor α in Equations (11) and (12) is set to 10. In each incremental learning stage, the feature projection layer is iteratively optimized for 1000 times using the new category samples and replayed prototypes. The SGD optimizer is used in this stage with a learning rate of 0.01.

### 4.3. Performance Comparison

This section provides a quantitative comparison and analysis of the proposed model with other state-of-the-art 5-way 5-shot FSCIL methods. Specifically, we conduct 5-way 5-shot few shot class incremental experiments on CIFAR-100 and *mini*ImageNet. The detailed experimental results are demonstrated in Table 1 and Table 2. We find that the proposed model outperforms other methods by approximately 3–5% in session 0 for the classification of 60 base classes on both datasets, primarily due to the effectiveness of the pre-training and meta-training procedure in generating robust features and reducing the correlations among prototypes of different classes. Overall, on CIFAR-100, the proposed model achieves a performance improvement of approximately 1% over other methods in the last session. However, on *mini*ImageNet, the results are always the best in each session. The reason lies in that *mini*ImageNet is more complex, which makes the quality of sample features in each task relatively higher. So the model can be adaptive to new categories after efficient pre-training and meta-training. Consequently, the proposed incremental-training stage aims to adjust its fitting ability to distinguish the prototypes of different classes as incremental classes accumulate. Hence, it achieves an improvement of approximately 2% in session 8 on *mini*ImageNet.

### 4.4. Qualitative Analysis

**Figure 7:**
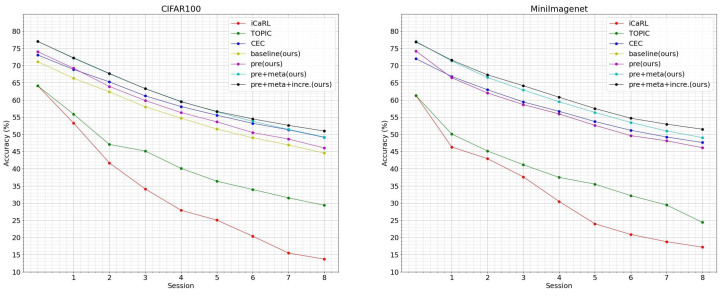
The performance curve of different typical 5-way 5-shot FSCIL methods in each session on CIFAR-100 and *mini*ImageNet.

We first qualitatively analyze the trend of performance in different sessions of various methods, as shown in Figure 7. The keys to improve the performance of class incremental learning lie in two aspects: how to train a robust feature extractor that can achieve high classification accuracy in session 0, and how to mitigate forgetting of old knowledge and facilitate learning of new knowledge to alleviate the decline in performance. Notably, the model can even achieve an improvement of 4–5% with only an efficient pre-training stage compared with typical methods iCaRL [10] and TOPIC [38], which benefits from the augmentations utilized in the pre-training stage. Moreover, the meta-training stage can further bring large improvements to the performance. Compared with CEC [36], our method equipped with pre-training and meta-training achieves higher accuracy in the first few sessions, which is because the implicit restriction of prototypes during meta-training helps distinguish the base samples. However, when encountered with novel samples in the incremental stage, the model cannot directly adapt to new classes and the accuracy drops a lot, so it achieves similar performance to CEC [36] in the last session. Based on the former two stages, incremental training further helps accommodate to novel classes and slows down the decline trend of performance. Therefore, it achieves an improvement of 1–4% compared with CEC [36].

**Figure 8:**
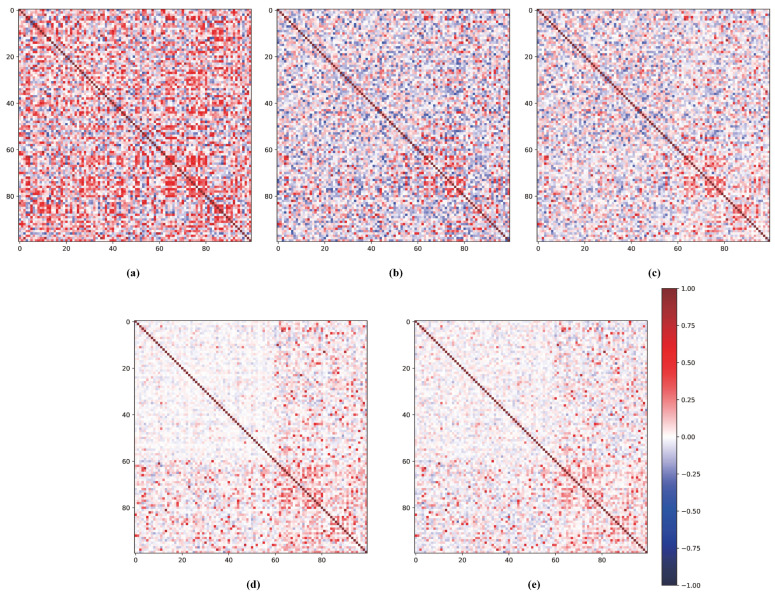
The visualization of confusion matrix with different methods on CIFAR-100. (**a**) pre-train; (**b**) pre-train+meta-train ($\zeta_{1}$); (**c**) pre-train+meta-train+incremental-train ($\zeta_{1}$); (**d**) pre-train+meta-train ($\zeta_{2}$); (**e**) pre-train+meta-train+incremental-train ($\zeta_{2}$).

Furthermore, we visualize in Figure 8 the confusion matrix of feature prototypes under different combinations of pre-training, meta-training, and incremental training on the CIFAR-100 dataset. In (a), where only pre-training is performed, there is a significant overlap of correlations between different feature prototypes in the confusion matrix (represented by dense dark red and dark blue blocks). In (b), (c), (d), and (e), the meta-training stage largely alleviates these correlations among different feature prototypes through learning from a series of sampled few shot tasks, which are represented by lighter red and blue blocks. Moreover, (d) and (e) respectively improve the ζ function on the basis of (b) and (c), reducing the overlap between feature vectors of different categories obtained by the meta-training model (also presented by lighter colors of blocks). Finally, (c) and (e) perform further incremental training on the basis of (b) and (d), respectively, which reduces the overlap between the later 40 new categories and all other categories in the incremental stage (i.e., the overall color of the other blocks except for the 60×60 block in the upper-left corner of the figure becomes lighter and the angle is closer to 90 degrees). In general, the qualitative analysis in Figure 8 fully demonstrates the rationality and effectiveness of the three stages of our proposed EPRC.

### 4.5. Ablation Study

In this section, we first conduct a series of 5-way 5-shot ablation FSCIL experiments with different combinations of training stages on CIFAR-100 and *mini*Imagenet to demonstrate the rationality and effectiveness of our EPRC. The corresponding results are listed in Table 3 and Table 4.

During the pre-training stage, the model is trained to learn rich knowledge from base classes. Strong augmentations in pre-training promote adequate information mining of base training data and boost discriminative representations of base classes. Furthermore, the meta-training stage improves the performance of the model by 3–5% on both benchmark datasets. Equipped with incremental training, the improvement of performance in the early sessions is not that significant. The reason lies in that the number of incremental classes is relatively small, and the final accuracy is still dominated by the base classes. The experimental results in the later sessions of the incremental learning stage show that it brings over 1.5% improvements. With respect to different nonlinear transformation functions, $\zeta_{2}$ plays a significant auxiliary role in meta-training, reducing the confusion among sample features of different classes. $\zeta_{2}$ aims to alter the distribution of different prototypes in feature space, thus making space for novel prototypes calibration without hindering discrimination of base samples. Similarly, comparing the experimental results of using $\zeta_{1}$ and $\zeta_{2}$ for meta-training, although there are no significant differences between performances in the early sessions, when the incremental classes accumulate (i.e., the later sessions of the incremental learning stage), the model trained with $\zeta_{2}$ achieves about 1% higher overall performance than that trained with $\zeta_{1}$. In summary, on the basis of pre-training with augmentations, our EPRC not only alleviates the confusion between base prototypes via meta-training, but also takes relationships of all base and novel prototypes into consideration, thus mitigating the decline trend of performance when classes accumulate.

In addition, the number of dimensions in the feature space not only determines how much information it can contain, but also affects the maximum number of orthogonal vectors that can be accommodated within it. Therefore, studying the impact of feature dimensions is of significant importance. Given that the dimension of features generated by ResNet-12 is 640, we present a detailed analysis of the performance with projected feature of different dimensions (i.e., 32, 64, 128, 256 and 512) on CIFAR-100, as shown in Table 5. Constrained by the scale of datasets, we study the incremental learning within only 100 classes. The results in Table 5 indicate that, when the feature dimension exceeds the number of classes, the variation in classification performance with an increase in the number of classes is negligible. However, when the number of feature dimension is smaller than that of the classes, the classification performance significantly declines. This observation implies that, if feature dimension is much smaller, there will be severe correlations among different prototypes, which limits the performance of our EPRC.

Furthermore, we also study the effect of scaling factor α to performances. As shown in Table 6, we choose different α values (i.e., 0.1, 1, 5, 10, 20, 50) and conduct 5-way 5-shot experiments on CIFAR-100. Theoretically, when α is smaller, the distribution of initial prediction becomes smoother, which requires the model to output sharper distribution in the training procedure. However, this is difficult in few shot scenarios. Large α can amplify the distinction of different samples and then make the model more sensitive to samples via meta-training, which is significant when encountered by few shot tasks. The experimental results show that EPRC performs well when α is greater than 5 and achieves the best performance when α is 10. Thus, we set α to 10 in our EPRC to conduct FSCIL.

To summarize, the combination of pre-training, meta-training, and incremental training in our EPRC gradually advances performances of FSCIL. Pre-training and meta-training not only boost the accuracy on base classes by a large margin, but also enhance the generalization ability pf our model to adapt to novel classes. Incremental training probes into the relations among prototypes of all encountered base and novel classes and adjusts them to be as distinguishable as possible, thus avoiding confusion in similarity measurement and then slowing down the decline of performance with quantity of sessions increasing.

## 5. Conclusions

In this paper, we propose an efficient feature replay and calibration (EPRC) method for few shot class incremental learning. As a preliminary, efficient pre-training equipped with rotation and random mix-up as an auxiliary supervision brings large improvement to subsequent training or evaluation. From the perspective of the overfitting problem, the meta-training stage with a series of sampled pseudo few shot tasks helps the model quickly adapt to novel few shot incremental tasks. In this procedure, the feature projection layer trained together with the pre-trained backbone aims to calibrate the prototypes into less correlated ones by means of an implicit constraint of similarities. With regard to the catastrophic forgetting problem, prototype storage and replay play an explicit but important role in memorizing old knowledge. To further improve the discrimination of different prototypes, we incorporate an explicit restriction into the incremental stage. The qualitative and quantitative experimental results on two benchmarks demonstrate that each stage in EPRC is effective and rational. For future work, cross-modal information can be utilized in the incremental stage to help enrich the old information, e.g., prompt-based methods [54].

## Figures and Tables

**Table 1:** Comparison with the state-of-the-art 5-way 5-shot FSCIL methods on CIFAR-100.

Methods	Accuracy in Each Session (%)								
	0	1	2	3	4	5	6	7	8
iCaRL [10]	64.10	53.28	41.69	34.13	27.93	25.06	20.41	15.48	13.73
NCM [53]	64.10	53.05	43.96	36.97	31.61	16.73	21.23	16.78	13.54
TOPIC [38]	64.10	55.88	47.07	45.16	40.11	36.38	33.96	31.55	29.37
SPPR [35]	64.10	65.86	61.36	57.45	53.69	50.75	48.58	45.66	43.25
CEC [36]	73.07	68.88	65.26	61.19	58.09	55.57	53.22	51.34	49.14
MetaFSCIL [37]	74.50	70.10	66.84	62.77	59.48	56.52	54.36	52.56	49.97
Data-free Replay [34]	74.40	70.20	66.54	62.51	**59.71**	56.58	54.52	52.39	50.14
EPRC	**77.02**	**72.25**	**67.70**	**63.29**	59.50	**56.67**	**54.51**	**52.62**	**50.98**

There are 60 base categories in session 0 and 5 new categories in each session 1–8. The best results are in bold.

**Table 2:** Comparison with the state-of-the-art 5-way 5-shot FSCIL methods on *mini*ImageNet.

Methods	Accuracy in each session (%)								
	0	1	2	3	4	5	6	7	8
iCaRL [10]	61.31	46.32	42.94	37.63	30.49	24.00	20.89	18.80	17.21
NCM [53]	61.31	47.80	39.30	31.90	25.70	21.40	18.70	17.20	14.17
TOPIC [38]	61.31	50.09	45.17	41.16	37.48	35.52	32.19	29.46	24.42
SPPR [35]	61.45	63.80	59.53	55.53	52.50	49.60	46.69	43.79	41.92
CEC [36]	72.00	66.83	62.97	59.43	56.70	53.73	51.19	49.24	47.63
MetaFSCIL [37]	72.04	67.94	63.77	60.29	57.58	55.16	52.79	50.79	49.19
Data-free Replay [34]	71.84	67.12	63.21	59.77	57.01	53.95	51.55	49.52	48.21
EPRC	**76.87**	**71.58**	**67.31**	**64.15**	**60.80**	**57.52**	**54.69**	**52.96**	**51.50**

There are 60 base categories in session 0 and 5 new categories in each session 1–8. The best results are in bold.

**Table 3:** Ablation study of 5-way 5-shot FSCIL with different combinations of training stages on CIFAR-100.

Methods	Accuracy in Each Session (%)								
	0	1	2	3	4	5	6	7	8
Baseline	71.10	66.32	62.36	58.01	54.71	51.56	49.03	46.92	44.57
Pre	74.04	69.23	63.92	59.85	56.34	53.65	50.53	48.65	46.05
Pre + Meta ($\zeta_{1}$)	75.87	70.82	66.09	61.60	57.81	54.67	51.97	49.47	47.10
Pre + Meta + Incre. ($\zeta_{1}$)	76.00	71.29	66.36	62.35	58.77	55.92	53.50	50.97	49.02
Pre + Meta ($\zeta_{2}$)	**77.03**	72.15	67.61	**63.32**	**59.58**	56.54	53.82	51.44	49.22
Pre + Meta + Incre. ($\zeta_{2}$)	77.02	**72.25**	**67.70**	63.29	59.50	**56.67**	**54.51**	**52.62**	**50.98**

There are 60 base categories in session 0 and 5 new categories in each session 1–8. The best results are in bold. Baseline: simply pre-train without augmentations; Pre: pre-train with augmentations; Meta: meta-train; Incre.: incremental-train.

**Table 4:** Ablation study of 5-way 5-shot FSCIL with different combinations of training stages on *mini*Imagenet.

Methods	Accuracy in Each Session (%)								
	0	1	2	3	4	5	6	7	8
Baseline	71.02	63.72	58.60	55.64	53.16	50.72	47.50	45.62	44.42
Pre	74.24	66.46	62.04	58.66	55.95	52.63	49.63	48.13	46.13
Pre + Meta ($\zeta_{1}$)	76.92	71.15	66.00	61.69	58.30	54.72	51.79	49.16	46.93
Pre + Meta + Incre. ($\zeta_{1}$)	**77.08**	**71.60**	67.24	63.79	60.31	56.81	54.16	52.34	50.38
Pre + Meta ($\zeta_{2}$)	77.03	71.34	66.54	62.91	59.48	56.38	53.46	50.99	49.07
Pre + Meta + Incre. ($\zeta_{2}$)	76.87	71.58	**67.31**	**64.15**	**60.80**	**57.52**	**54.69**	**52.96**	**51.50**

There are 60 base categories in session 0 and 5 new categories in each session 1–8. The best results are in bold. Baseline: simply pre-train without augmentations; Pre: pre-train with augmentations; Meta: meta-train; Incre.: incremental-train.

**Table 5:** Ablation study of 5-way 5-shot FSCIL with different feature dimensions on CIFAR-100.

Dimension	Accuracy in Each Session (%)								
	0	1	2	3	4	5	6	7	8
32	74.70	69.13	64.44	59.64	56.02	52.96	50.34	47.75	45.13
64	76.50	71.55	65.96	62.05	58.15	55.85	53.13	50.13	47.95
128	76.83	71.93	66.46	62.94	59.30	56.55	54.20	52.34	49.55
256	77.01	72.03	67.56	62.95	**59.60**	56.63	54.50	52.20	50.24
512	**77.02**	**72.25**	**67.70**	**63.29**	59.50	**56.67**	**54.51**	**52.62**	**50.98**

There are 60 base categories in session 0 and 5 new categories in each session 1–8. The best results are in bold.

**Table 6:** Ablation study of 5-way 5-shot FSCIL with different α on CIFAR-100.

α Value	Accuracy in Each Session (%)								
	0	1	2	3	4	5	6	7	8
0.1	69.53	63.86	59.57	54.25	50.95	46.14	43.84	41.69	37.54
1	71.95	67.05	62.94	58.51	55.40	51.85	49.09	47.07	44.20
5	74.68	70.91	65.85	61.55	58.74	55.89	53.13	50.94	48.05
10	**77.02**	**72.25**	**67.70**	**63.29**	**59.50**	**56.67**	**54.51**	**52.62**	**50.98**
20	75.98	71.25	66.94	62.81	59.12	56.39	53.70	51.32	49.24
50	75.23	70.94	66.63	62.64	59.08	56.29	53.79	51.65	49.40

There are 60 base categories in session 0 and 5 new categories in each session 1–8. The best results are in bold.

## Data Availability

CIFAR-100: https://www.cs.toronto.edu/~kriz/cifar.html (accessed on 10 March 2021); *mini*Imagenet: https://github.com/yaoyao-liu/mini-imagenet-tools (accessed on 10 March 2021).
